# Complete genome analysis of *Gluconacetobacter xylinus* CGMCC 2955 for elucidating bacterial cellulose biosynthesis and metabolic regulation

**DOI:** 10.1038/s41598-018-24559-w

**Published:** 2018-04-19

**Authors:** Miao Liu, Lingpu Liu, Shiru Jia, Siqi Li, Yang Zou, Cheng Zhong

**Affiliations:** 1 Key Laboratory of Industrial Fermentation Microbiology, Ministry of Education, Tianjin University of Science and Technology, Tianjin, 300457 P. R. China; 2 Tianjin Jialihe Livestock Group Co., Ltd, JinWei Road, Beichen District, Tianjin, 300402 P. R. China

## Abstract

Complete genome sequence of *Gluconacetobacter xylinus* CGMCC 2955 for fine control of bacterial cellulose (BC) synthesis is presented here. The genome, at 3,563,314 bp, was found to contain 3,193 predicted genes without gaps. There are four BC synthase operons (*bcs*), among which only *bcs*I is structurally complete, comprising *bcs*A, *bcs*B, *bcs*C, and *bcs*D. Genes encoding key enzymes in glycolytic, pentose phosphate, and BC biosynthetic pathways and in the tricarboxylic acid cycle were identified. *G. xylinus* CGMCC 2955 has a complete glycolytic pathway because sequence data analysis revealed that this strain possesses a phosphofructokinase (pfk)-encoding gene, which is absent in most BC-producing strains. Furthermore, combined with our previous results, the data on metabolism of various carbon sources (monosaccharide, ethanol, and acetate) and their regulatory mechanism of action on BC production were explained. Regulation of BC synthase (Bcs) is another effective method for precise control of BC biosynthesis, and cyclic diguanylate (c-di-GMP) is the key activator of BcsA–BcsB subunit of Bcs. The quorum sensing (QS) system was found to positively regulate phosphodiesterase, which decomposed c-di-GMP. Thus, in this study, we demonstrated the presence of QS in *G. xylinus* CGMCC 2955 and proposed a possible regulatory mechanism of QS action on BC production.

## Introduction

Bacterial cellulose (BC), naturally produced by several species of *Acetobacter*, is a strong and ultra-pure form of cellulose^1^. It has the same chemical structure as plant cellulose but is devoid of hemicellulose and lignin^2^. Until now, many researchers have endeavoured to control BC production to realise its potential applications. Most applications have been implemented by optimizing the carbon source and culture conditions^3,4^. Additionally, functionalisation or modification of BC has mainly been achieved via chemical or mechanical modifications of the cellulose matrix^5,6^. Nowadays, synthetic biology enables model microorganisms to be easily reprogrammed with modular DNA code to perform a variety of new tasks for useful purposes^7^. This approach may allow for more finely tuned control over BC synthesis by gene regulation. Nonetheless, only a few attempts at genetic engineering have been made for BC-producing bacteria, and the toolkit for genetic modification has hardly been described^8^. Thus, it is essential for researchers to gain genomic insights into various BC-producing strains.

So far, several BC-producing *Gluconacetobacter* (*Komagataeibacter*) species have been sequenced, and the genomes of *K. xylinus* E25^9^, *K. medellinensis* NBRC 3288 (ref.^10^), *K. nataicola* RZS01^11^, and *Gluconobacter oxydans* DSM 3504 were sequenced completely. *Gluconacetobacter xylinus* (formerly *Acetobacter xylinum*) is one of the most commonly studied species because of its ability to produce relatively large amounts of BC from a wide range of carbon and nitrogen sources in liquid culture^3,4,12^ and has become the most popular strain so far for manufacturing of various materials, including paper^13^, food packaging^14^, medical materials^15^, and cell culture^16^. It is difficult to obtain high productivity by means of *G. xylinus* in a large-scale fermentation system owing to its low yield under agitated culture. Nonetheless, BC secreted by *G. xylinus* has unique properties including high quality in terms of mechanical strength, water-holding capacity, crystallinity, biodegradability, and biocompatibility^17^. *G. xylinus* CGMCC 2955 originates from the fermentation substrates of vinegar^18^. It has been studied as a model organism for BC production for decades and has been successfully applied to production of wound dressings^19,20^. In the current study, we present a complete genome sequence of *G. xylinus* CGMCC 2955 to provide background information for genetic engineering so that precise control of BC biosynthesis can be achieved on the basis of the metabolic pathway that we proposed previously^3^. A comparison of the arrangement and composition of BC synthase operons (*bcs*) with those of other BC-producing strains was performed. Furthermore, the relation between the *bcs* operon and quorum sensing (QS) system – a widely studied system for population regulation – is discussed.

## Results

Sequencing showed that the genome is 3,563,314 bp long with GC content of 63.29%. One scaffold was obtained without gaps (Table 1). Gene prediction and annotation of the *G. xylinus* CGMCC 2955 genome resulted in 3,193 predicted genes. Amongst 117 non-coding RNAs, there are 45 rRNAs, 57 tRNAs, and 15 other RNAs. In the repeat DNAs, simple repeats turned out to be the most abundant repeat families, accounting for 0.51% of the *G. xylinus* CGMCC 2955 genome, followed by small RNA, accounting for 0.20% of the genome. Non–long terminal repeats were also identified in the genome and contain 13 short interspersed nuclear elements and nine long interspersed nuclear elements. Besides, six DNA transposons (five DNA/hAT-Ac specimens and one DNA/TcMar-Tigger) were detected. No long terminal repeats or satellites were observed.

**Table 1:** General features of *G. xylinus* CGMCC 2955 genome.

Genome size (bp)	3,563,314
Gap N (bp)	0
GC content (%)	63.29
Total genes	3,139
Non-coding RNA	117
rRNA	45
tRNA	57
others	15
Repeat DNAs	
SINEs	13
LINEs	9
LTR	0
DNA elements	6
Small RNA	41
Satellites	0
Simple repeats	409
Low complexity	38

SINEs: short interspersed elements.

LINEs: long interspersed nuclear elements.

LTR: long terminal repeat.

**Figure 1:**
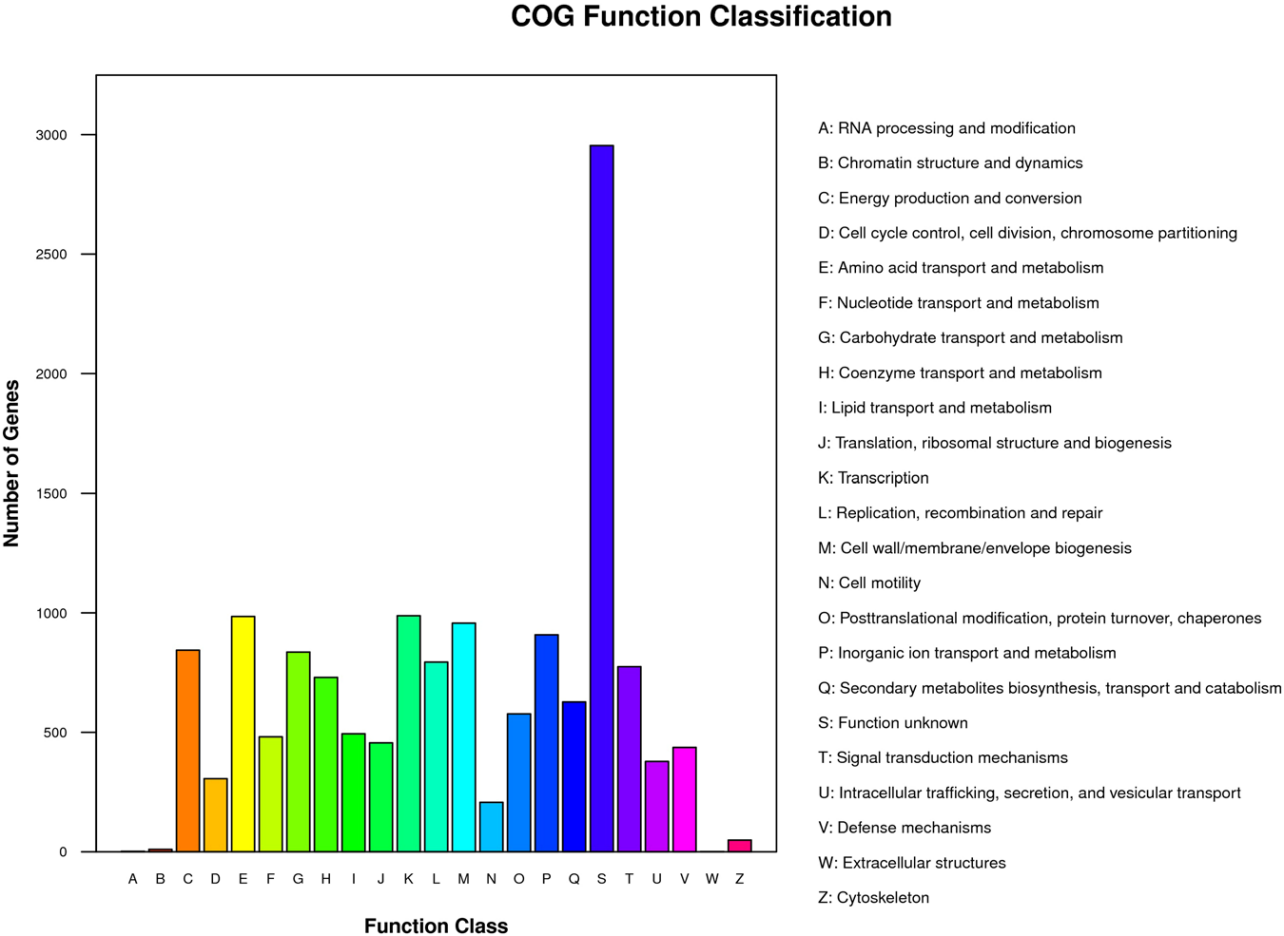
Cluster of orthologous groups (COG) classification of protein functions.

The cluster of orthologous groups (COG) function-based classification of *G. xylinus* CGMCC 2955 genes revealed 23 functional groups, in which 19.96% of the genes are functionally uncharacterised (Fig. 1). The second and third largest functional groups were ‘transcription’ and ‘amino acid transport and metabolism’; they consisted of 988 and 985 genes, respectively.

### Carbon source metabolism

A total of 50 genes of transporters, 13 genes of symporters, and 50 genes of permeases were identified in the genome of *G. xylinus* CGMCC 2955. They are responsible for the transport of sugars (e.g. glucose, arabinose, and galactose), sugar acids (e.g. gluconate, α-ketoglutarate, and galactonate), amino acids, spermidines, vitamin B12, phosphate, metal ions, and lipopolysaccharide.

*G. xylinus* CGMCC 2955 is capable of producing BC from several carbon sources^3^. Generally, glucose (Glc) is metabolised through the Embden–Meyerhof–Parnas (EMP) pathway, which has proven to be incomplete in most BC-producing stains because of the lack of a gene encoding phosphofructokinase (PFK; EC 2.7.1.11)^11,21,22^. Of note, according to the gene sequence analysis, the *G. xylinus* CGMCC 2955 genome possesses a gene encoding phosphofructokinase B (*pfkB*, CT154_09360), while there is no gene encoding phosphofructokinase A (*pfkA*). The other two key enzymes are glucokinase (GK) and pyruvate kinase (PK), whose encoding gene locus tags are CT154_11525 and CT154_15810, respectively. The pentose phosphate pathway (PPP) is another effective glucose metabolic pathway in *G. xylinus*. In the *G. xylinus* CGMCC 2955 genome, genes encoding glucose-6-phosphate dehydrogenase (*6pgd*, CT154_14710), glucose-6-phosphate 1-dehydrogenase (*6pgd-1*, CT154_14205 and CT154_05345), 6-phosphogluconolactonase (*6pgl*, CT154_14190), 6-phosphogluconate dehydrogenase (*6pgad*, CT154_14210), ribose-5-phosphate isomerase A (*rpi A*, CT154_14185), ribulose-phosphate 3-epimerase (*rpe*, CT154_13290), transketolase (*tkt*, CT154_07695, CT154_08455, CT154_12910, and CT154_14220), and transaldolase (*tsd*, CT154_07690 and CT154_14215) were identified. Gluconic acid (GlcA) is an important by-product of BC production in *G. xylinus*^3,23^. It was found to be produced from glucose by glucose dehydrogenase (*gd*, CT154_01250 and CT154_06665) and can be converted to 6-phosphogluconate (GlcA6P) by gluconokinase (GntK). There are two genes encoding GntK (CT154_14180 and CT154_03305), and the latter was hypothesised to encode a thermosensitive GntK. The tricarboxylic acid cycle (TCA) has three key enzymes – citrate synthase (CT154_06205), isocitrate dehydrogenase (CT154_11710 and CT154_00570), and 2-oxoglutarate dehydrogenase (CT154_14680) – and the genes coding for these enzymes are all present in *G. xylinus* CGMCC 2955. Fructose (FRU) and glycerol (GLY) can considerably contribute to a BC yield just as glucose can^3^. Via catalysis by fructokinase (*frk*, CT154_06355), fructose was found to be converted to fructose-6-phosphate (F6P). When glycerol was utilised for BC production, it was firstly converted to glycerol 3-phosphate (GLY3P) by glycerol kinase (*glyk*, CT154_10145 and CT154_10550), and then was transformed into dihydroxyacetone phosphate (DHAP) and glyceraldehyde-3-phosphate (GAP) by glycerol-3-phosphate dehydrogenase (G3PD) and triosephosphate isomerase (TPI), entering the EMP pathway and PPP. Their encoding gene locus tags were found to be CT154_10150 and CT154_05740, respectively.

**Figure 2:**
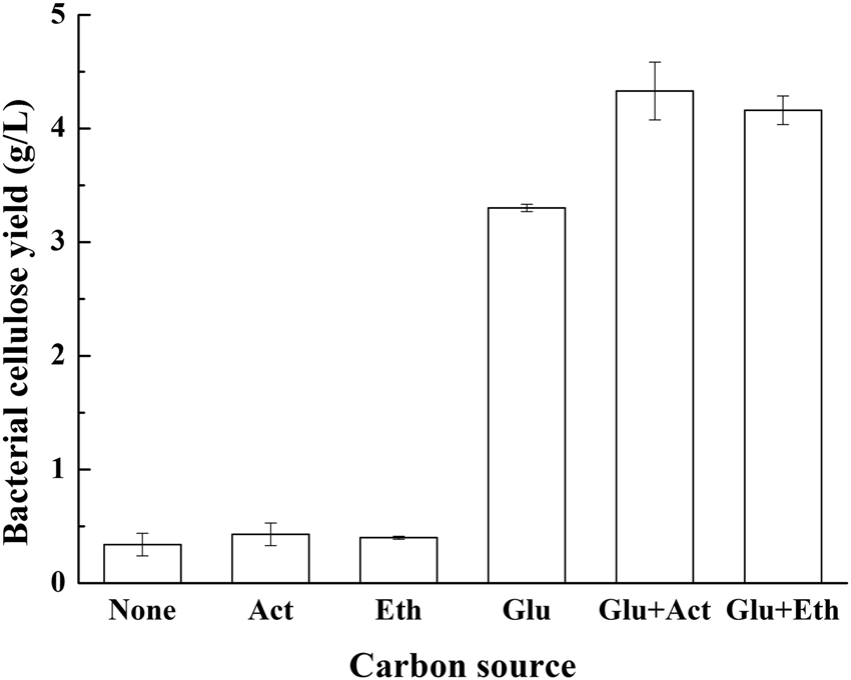
BC production from ethanol and acetate as the sole or supplementary carbon sources. (None: no carbon source, Act: acetate, Eth: ethanol, Glu: glucose).

When ethanol (ETH) or acetate (AC) served as a sole carbon source for BC production, as shown in Fig. 2, both contributed to a BC production of 0.4 g/l. Glucose was then added as the main carbon source, and either ethanol or acetate were used as supplementary carbon sources. As compared to glucose as the sole carbon source, the ethanol and acetate supplementation increased the BC yield to 1.26-fold and 1.31-fold, respectively. When ethanol is added as the supplementary carbon source, *Acetobacter xylinum* BPR 3001 A can synthesise BC with a higher yield^24^. In BPR 3001 A, ethanol is first converted to acetaldehyde by alcohol dehydrogenase (ADH), and then acetaldehyde is converted to acetate under the action of aldehyde dehydrogenase (ALDH). After that, acetyl-coenzyme A (ACCoA) is produced from acetate. In the genome of *G. xylinus* CGMCC 2955, the gene sequences of *Adh* (CT154_02385, CT154_08135, CT154_09950, CT154_11925, and CT154_13530) and *Aldh* (CT154_00245, CT154_02130, CT154_06670, and CT154_12865) were found to be available. In *Escherichia coli*, acetate can be utilised as a sole carbon source in the phosphotransacetylase-acetate kinase pathway (Pta-Ack), in which phosphate acetyltransferase (PATs) and acetate kinase (ACK) are required^25^, encoded by CT154_12395 and CT154_15860 in *G. xylinus* CGMCC 2955.

### Energy metabolism

The BC yield enhancement by ethanol and acetate supplementation has been reported to be related to adenosine triphosphate (ATP) production^24^. There are two patterns for ATP production: substrate level phosphate production through glucose metabolism and oxidative phosphate production via a reduced form of nicotinamide-adenine dinucleotide (NADH) (or reduced flavin adenine dinucleotide, FADH_2_) generated by glucose metabolism and transferred to electron acceptors. Glucose metabolism is the main energy source of *G. xylinus* CGMCC 2955.

Under aerobic conditions, oxygen is the electron acceptor from NADH and FADH_2_ through the electron transport chain. Under these conditions, energy production is mainly based on oxidative phosphorylation, supplemented by substrate level phosphorylation^26^. Under anaerobic culture conditions, nitrate acts as the electron acceptor in *Enterobacter* sp. FY-07, which is able to produce BC with a high yield under anaerobic conditions. It is reduced to nitrite by accepting the electrons transferred through the electron transport chain, and nitrite is then reduced to ammonia^27^. Nitrate reductase and nitrite reductase are involved in this process, and in the present study, only a nitrite reductase gene (CT154_11275) was identified in the *G. xylinus* CGMCC 2955 genome. Moreover, genes encoding ADH (CT154_02385, CT154_05910, CT154_08135, CT154_09950, CT154_11925, and CT154_13530) and lactate dehydrogenase (*ldh*, CT154_01275 and CT154_10640) were identified in the *G. xylinus* CGMCC 2955 genome. They are responsible for the production of NADH when oxygen supply is deficient. This finding suggested that *G. xylinus* CGMCC 2955 has the potential to produce sufficient energy in hypoxic environments.

### BC synthesis

In *G. xylinus*, glycolytic intermediate glucose-6-phosphate is the origin of substrate synthesis for BC production. It is isomerised to glucose-1-phosphate (G1P) by phosphoglucomutase (PGM; encoded by CT154_06830), which then reacts with UTP, forming uridine-5′-phosphate-α-D-glucose (UDPG) under the action of UDP-glucose pyrophosphorylase (UGPase; encoded by CT154_06835)^28^. Finally, cellulose is synthesised by a BC synthase (Bcs) complex containing subunits BcsA, BcsB, BcsC, and BcsD, which are encoded by three (*bcsAB*, *bcsC*, and *bc*s*D*) or four (*bcsA*, *bcsB*, *bcsC*, and *bcsD*) genes^11,29^.

The Bcs complex spans the outer and inner cell membranes to synthesise and extrude glucan chains, which are then assembled into fibrils that are further assembled into a ribbon^30^. BcsA is the catalytic subunit of the Bcs complex^28,31^. BcsB is an auxiliary subunit that interacts with BcsA via its C-terminal transmembrane helix and regulates BC synthesis by interacting with cyclic diguanylate (c-di-GMP)^32^. The BcsA–BcsB complex is sufficient for BC synthesis and translocation^31^. BcsC is believed to play important roles in cell membrane pore formation for BC secretion^32^. BcsD may control the crystallisation of cellulose into nanofibrils^5^. There can be one or several *bcs* operons in a single organism, and the sequence identity of genes encoding the same subunits may not be that high. Moreover, the composition and arrangement of *bcs* operons are diverse in the same or two different organisms. Additionally, among the *bcs* operons in the same genome, only one or some of the operons perform key functions in BC synthesis^33^.

**Figure 3:**
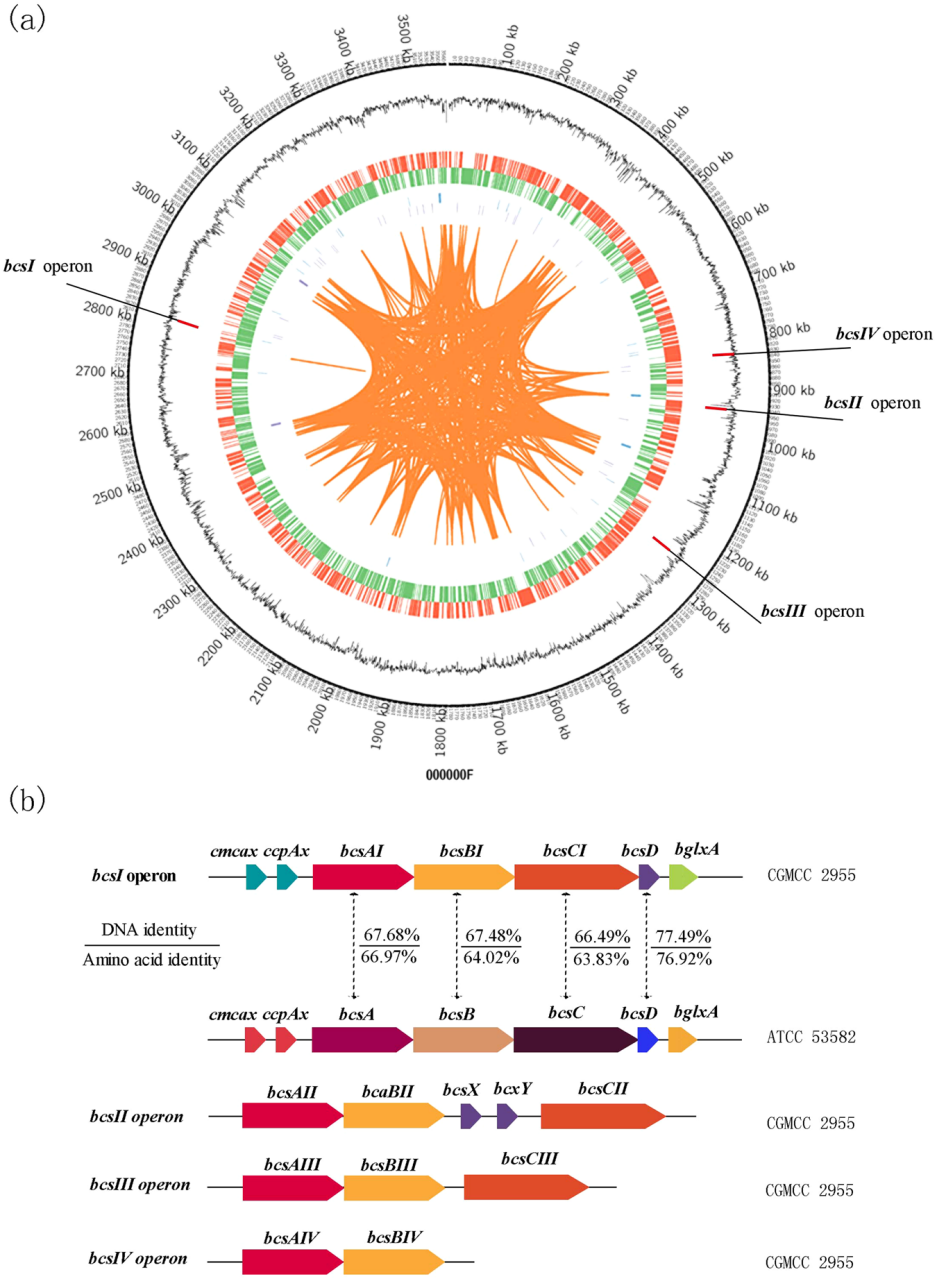
An overview of the *G. xylinus* CGMCC 2955 genome. (**a**) The circle represents (from outside in): circle 1, genome; circle 2, G + C content; circle 3, genes on forward (red) and reverse (green) strands; circle 4, distribution of genes encoding non-coding RNAs; circle 5, long terminal repeats in the genome. (**b**) The arrangement of *bcs* operons and sequence alignment of the *bcs* I operon and *G. xylinus* ATCC 53582 *bcs* operon.

In the *G. xylinus* CGMCC 2955 genome, a total of four *bcs* operons were identified. As shown in Fig. 3, their compositions were different among the operons. Operon *bcs* I was the only structurally complete one, containing *bcsA* (CT154_12695), *bcsB* (CT154_12690), *bcsC* (CT154_12685), and *bcsD* (CT154_12680). In addition to the above genes, *cmcax* (CT154_12705) and *ccpax* (CT154_12700) were identified upstream, and the *bglxA* (CT154_12675) gene was located downstream in operon *bcs I*. Gene *cmcax* encodes endo-β-1,4-glucanase (CMCax). When antibodies to recombinant CMCax are added to the culture medium, the formation of cellulose fibre is severely inhibited^34^. The CcpAx protein (also known as ORF-2), encoded by *ccpax*, functions as a mediator of protein–protein interactions and is important for localisation of the Bcs complex to the cell membrane. The *bglxa* gene encodes a β-glucosidase. CMCax and β-glucosidase both can hydrolyse tangled glucan chains when there is a failure in chain arrangement and are both crucial for BC synthesis^35^. Operons *bcs* II and *bcs* III consist of *bcsA*, *bcsB*, and *bcsC*, where BcsA and BcsB are encoded by one gene. Operon *bcs* II (*bcsA*, *bcsB*, and *bcsC*) was found to be encoded by CT154_04230 and CT154_04245, with *bcsX* (CT154_04235) and *bcsY* (CT154_04240) in the middle of *bcsB* and *bcsC*. The products of *bcsX* and *bcsY* have not yet been characterised although Bcs Y was predicted to function as a transacylase, participating in the production of acetyl cellulose or similarly modified polysaccharides^36^. Operon *bcs* III (*bcsA*, *bcsB*, and *bcsC*) turned out to be encoded by CT154_05660 and CT154_05665. Operon *bcs* IV uniquely contains only *bcsA* and *bcsB* (CT154_03760).

Sequence alignment and analysis of *bcsA* and *bcsB* in the four operons indicated that the identity was very low between them (Table 2). Operon *bcs* I was found to be structurally the same as the sole *bcs* operon in *G. xylinus* ATCC 53582. The sequence alignment of each gene is presented in Fig. 3b. It suggests that *bcsA* in *bcs* I (*bcsAI)* genes in *G. xylinus* CGMCC 2955 and *G. xylinus* ATCC 53582 share 67.68% and 66.97% nucleotide sequence identity and amino acid sequence identity, respectively. For *bcsBI*, the respective identity values were 67.48% and 64.02%. For *bcsCI*, the respective identity values were 66.49% and 63.83%. For *bcsDI*, the respective identity levels were 77.49% and 76.92%. There is only one *bcs* operon in *G. xylinus* ATCC 53582. The high identity of operon *bcs* I sequences between *G. xylinus* CGMCC 2955 and *G. xylinus* ATCC 53582, and its complete structure suggested that *bcs I* may be deeply involved in BC synthesis in *G. xylinus* CGMCC 2955. These results provide a foundation for genetic manipulation for research regarding which *bcs* operon is essential for BC synthesis in *G. xylinus* CGMCC 2955.

**Table 2:** sequence alignment of *bcs A-B* in four *bcs* operons of *G. xylinus* CGMCC 2955.

	*bcs* I A-B	*bcs* II A-B	*bcs* III A-B	*bcs* IV A-B
*bcs* I A-B	100	57.31	53.36	56.79
*bcs* II A-B	57.31	100	56.99	67.87
*bcs* III A-B	53.36	56.99	100	55.87
*bcs* IV A-B	56.79	67.87	55.87	100

### Regulation of BC synthesis by c-di-GMP and by the QS system

**Figure 4:**
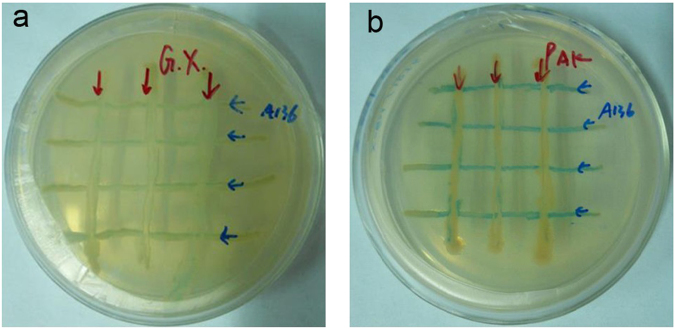
The reported vertical streaking method with bacterial biosensors. (**a**) *G. xylinus* CGMCC 2955. (**b**) *P. aeruginosa* PAK (positive control).

The BcsA–BcsB complex in Bcs is activated by c-di-GMP^37^. The latter is synthesised from guanosine triphosphate (GTP) by diguanylate cyclase (DGC) and is degraded by two phosphodiesterases (PDEs), thereby regenerating 5′-guanosine monophosphate (5′-GMP; that can be used for GTP synthesis) and forming a biosynthetic and degradative pathway: the c-di-GMP cycle^2,38^. In the *G. xylinus* CGMCC 2955 genome, there is only one *dgc* gene (CT154_08975), while there are several *pde* genes, including CT154_00885, CT154_01735 – CT154_01745, CT154_08940, CT154_08945, CT154_08975, and CT154_14645. Besides, CT154_05705 and CT154_14650 are hypothesised to encode DGC/PDE. These genes together control intracellular c-di-GMP levels. In *Gluconacetobacter intermedius*, *pde* expression was shown to be positively regulated by the GinI–GinR QS system^39,40^. The presence of a QS system can be determined by detection of chemical signals called autoinducers^41^. These signalling molecules are produced from common metabolites such as fatty acids, anthranilate, and S-adenosylmethionine^42–44^. In the current study, the presence of signalling molecules was identified by a vertical streaking agar well diffusion method. *Agrobacterium tumefaciens* A136 allows for detection of a broad range of acyl-homoserine lactones (AHLs) with acyl chain lengths from 4 to 12 carbons as well as 3-unsubstituted AHLs^45^. As shown in Fig. 4, the *A. tumefaciens* A136 biosensor turned blue in (a) and (b) indicating that *G. xylinus* CGMCC 2955 and *Pseudomonas aeruginosa* PAK both synthesise a QS AHL autoinducer signalling molecule (Fig. 4a,b)^46^. This finding revealed that *G. xylinus* CGMCC 2955 is capable of producing AHLs, and the latter are usually produced by the LuxI–LuxR QS system in gram-negative bacteria^47^. Genes *luxI* and *luxR* are normally homologs of *ginI* and *ginR*. Nevertheless, only the *lux*R (CT154_10285) gene was identified in *G. xylinus* CGMCC 2955.

## Discussion

To control the production of BC for different purposes, researchers manipulate the cell culture process. The carbon source was reported to be one of the most effective factors that contribute to differences in BC production and structural characteristics^12,48,49^. Zhong *et al*. revealed that *G. xylinus* CGMCC 2955 can produce BC from various carbon sources, including glucose, fructose, and glycerol^3^. Distinct BC network structures were obtained from these carbon sources, resulting from different metabolic processes, including BC production and cell growth rates. The metabolic network necessary to study the metabolism of different carbon sources has already been built^3^. In the present study, genome sequence analysis revealed the metabolic characteristics of *G. xylinus* CGMCC 2955. Most BC-producing strains do not possess the EMP pathway because they lack PFK activity^50^. Nevertheless, genome sequence analysis indicated that *G. xylinus* CGMCC 2955 has *pfkB* (CT154_09360), which encodes PFK II^51^. PFK I and II both convert fructose-6-phosphate (F6P) to fructose-1,6-diphosphate (F16P). In *E. coli*, ~90% of the activity is attributed to PFK I, a well-known allosteric enzyme. The remaining activity is PFK II^52^. Therefore, in contrast to other BC-producing microorganisms, *G. xylinus* CGMCC 2955 has a complete EMP pathway. Moreover, ~33% of glucose enters the PPP and 17% entered the TCA cycle in *G. xylinus* CGMCC 2955^3^. In the genome of *G. xylinus* CGMCC 2955, all genes encoding enzymes of the PPP were found, confirming that glucose could be metabolised by the PPP. Besides, flux analysis suggested that 92.85% of glucose is fluxed into gluconic acid^3^. Liu *et al*. speculated that gluconic acid can serve as a second carbon source when glucose is exhausted in *G. xylinus* CGMCC 2955^23^. The presence of gluconate kinase–encoding gene g*ntK* (CT154_14180, CT154_03305) – coupled with a flux analysis that indicated that more than a half of gluconic acid (52.83%) was converted to glucose-6-phosphate – suggested that gluconic acid may be utilised as a carbon source for cell growth and BC production through the PPP. This finding is consistent with our previous report showing that dry cell weight and the BC yield increased 2.55- and 14.17-fold, respectively, when gluconic acid was provided as the sole carbon source as opposed to no carbon source^53^.

When ethanol or acetate were used as the sole carbon source, neither of them could contribute to a considerable BC yield (Fig. 2). On the other hand, the genes encoding the enzymes of the Pta-Ack pathway are all present in *G. xylinus* CGMCC 2955. When *G. xylinus* CGMCC 2955 was incubated in a glucose-containing culture medium, both ethanol and acetate contributed to the increased BC yield. These results support other reports^24^. We believe that ethanol or acetate cannot be utilised as a substrate for BC production owing to the absence of a precursor for BC synthesis. Nevertheless, ethanol or acetate functioned as an energy source for ATP generation through the TCA cycle. Therefore, it was inferred that the promotion of BC production by supplementary ethanol or acetate can be attributed to ATP generation, which accelerated the BC synthesis pathway by inhibiting glucose-6-phosphate dehydrogenase activity^24^. Furthermore, the relation between ATP production and BC synthesis was recently studied in *Enterobacter* sp. FY-07, which is capable of synthesizing BC under both aerobic and anaerobic culture conditions^27^. The BC biosynthesis process in *Enterobacter* sp. FY-07 was found to be the same as that of *G. xylinus*. It manifested a high yield of BC even under anaerobic conditions, suggesting that oxygen was not directly involved in the BC synthesis reaction. Meanwhile, ATP was found to be necessary to activate glucose to UDPG, which is also essential for c-di-GMP biosynthesis. Our recent work indicates that *G. xylinus* CGMCC 2955 can produce more BC during hypoxia than under atmospheric and oxygen-enriched culturing conditions^53^. Further research on the effect that oxygen and energy generation have on BC production is a possible next project.

The Bcs complex is the key enzyme in BC synthesis. Four *bcs* operons were identified in the *G. xylinus* CGMCC 2955 genome. Up to three *bcs* operons in other BC-producing species have been reported (*G. xylinus* ATCC 23769 and *G. hansenii* ATCC 53582)^10,54,55^, where a high cellulose synthase copy number generally indicates contributions to high BC productivity^8^. Our phylogenetic analysis indicates that *bcs* II and *bcs* IV were the most closely related (Table 2) and possibly arose from duplication and subsequent translocation. With this information, we can study the function of different *bcs* operons, e.g. investigate whether operon *bcs* I, the only structurally complete *bcs* operon, functions alone. If the genes encoding Bcs subunits were deleted in *bcs* I, will other *bcs* operons act as candidate genes?

c-di-GMP, an activator of the BcsA–BcsB complex, is synthesised from GTP by DGC and is degraded by PDE^2^. In *G. intermedius*, the expression of *pde* was found to be positively regulated by the GinI–GinR QS system^39,40^. QS is a microbial cell-to-cell communication process that allows a group of bacterial cells to regulate their gene expression in unison, which is important for implementation of group behaviours such as bioluminescence^56^, virulence^57^, phenotypic heterogeneity^58^, genetic exchange^59^, and bacterial pathogenicity^60^. QS systems have been identified in both gram-negative and gram-positive bacterial species^61^. The LuxI–LuxR-type system is common among gram-negative QS bacteria, in which LuxR serves as both the cytoplasmic signalling molecule’s receptor and the transcriptional activator of the *lux* operon’s QS system, whereas LuxI works as the signalling molecule’s synthase^47,62,63^. In this study, Fig. 4 illustrates the presence of AHLs in *G. xylinus* CGMCC 2955. AHLs were synthesised by the LuxR–LuxI system. When the AHL signal reaches a threshold concentration, it is bound by LuxR, and this complex activates the transcription of a specific operon. Nonetheless, in the *G. xylinus* CGMCC 2955 genome, only *luxR* (CT154_10285) was identified. The absence of *luxI* suggests that the signalling molecule may be produced by other pathways instead of *luxI* or that the identity of the *lu*x*I* sequence in *G. xylinus* CGMCC 2955 with that in other microorganisms is too low for identification of *lu*x*I*.

**Figure 5:**
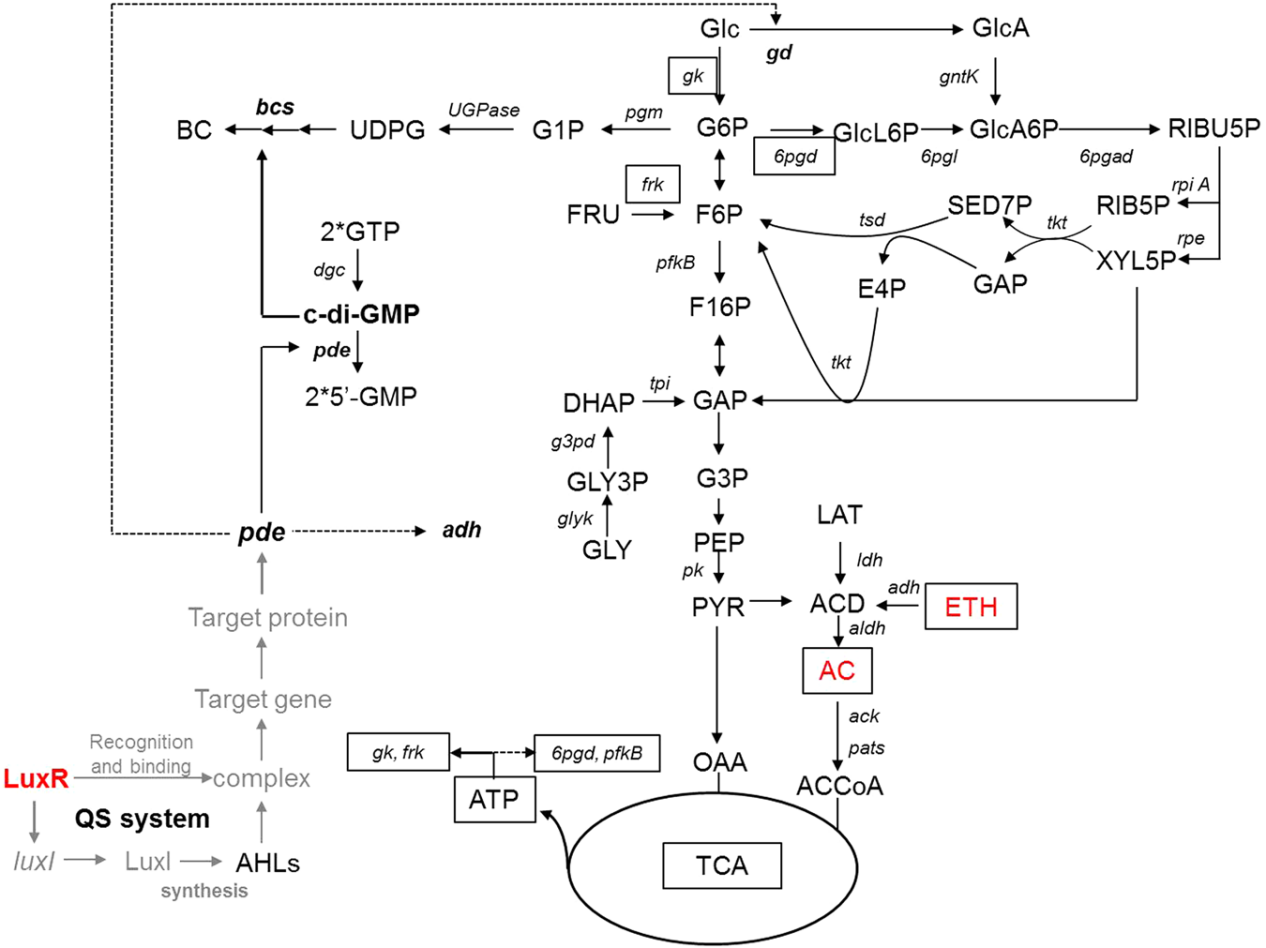
The metabolic and regulatory network of *G. xylinus* CGMCC 2955. Framed means regulation by ethanol and acetate addition, and boldfacing denotes regulation by the QS system. Gray means needing further examination. A solid line indicates that gene expression, metabolite synthesis, or metabolic pathway was activated; a dashed line means they were inhibited. (Abbreviations of genes. *6pgad*: 6-phosphogluconate dehydrogenase; *6pgd*: glucose-6-phosphate dehydrogenase; *6pgl*: 6-phosphogluconolactonase; *ack*: acetate kinase; *adh*: alcohol dehydrogenase; *aldh*: aldehyde dehydrogenase; *bcs*: bacterial cellulose synthase; *dgc*: diguanylate cyclase; *frk*: fructokinase; *g3pd*: glycerol-3-phosphate dehydrogenase; *gd*: glucose dehydrogenase; *gk*: glucokinase; *glyk*: glycerol kinase; *gntK*: gluconokinase; *ldh*: lactate dehydrogenase; *pats*: phosphate acetyltransferase; *pde*: phosphodiesterase; *pfkB*: phosphofructokinase B; *pgm*: phosphoglucomutase; *pk*: pyruvate kinase; *rpe*: ribulose-phosphate 3-epimerase; *tkt*: transketolase; *tpi*: triosephosphate isomerase; *tsd*: transaldolase; *UGPase*: UDP-glucose pyrophosphorylase), (Abbreviations of metabolites. AC: acetate; ACCOA: acetyl-coenzyme A; ACD: acetaldehyde; BC: bacterial cellulose; c-di-GMP: cyclic diguanylate; DHAP: dihydroxyacetone phosphate; E4P: erythrose 4-phosphate; ETH: ethanol; F16P: fructose-1,6-diphosphate; F6P: fructose 6-phosphate; FRU: fructose; G1P: glucose 1-phosphate; G3P: glyceraldehyde-3-phosphate; G6P: glucose 6-phosphate; GAP: glyceraldehyde-3-phosphate; Glc: glucose; GlcA: gluconate; GlcA6P: 6-phosphogluconate; GlcL6P: 6-phosphogluconolactone; GLY: glycerol; GLY3P: glycerol 3-phosphate; GMP: guanosine monophosphate; GTP: guanosine triphosphate; LAT: lactate; OAA: oxaloacetate; PEP: phosphoenol pyruvate; PYR: pyruvate; RIB5P: ribose-5-phosphate; RIBU5P: ribulose-5-phosphate; SED7P: sedoheptulose 7-phosphate; UDPG: uridine-5′-phosphate-α-D-glucose; XYL5P: xylulose-5-phosphate).

The regulation of the QS system by PDE involves the 89 aa protein GinA (target protein) in *G. intermedius*, where the production of GinA is induced by the QS system. A putative c-di-GMP phosphodiesterase, *pde* was shown to be GinA inducible and is involved in the repression of oxidative fermentation by the GinI–GinR QS system^40^. Therefore, because *luxI* and *luxR* are homologs of *ginI* and *ginR*, and c-di-GMP is an activator of the BcsA–BcsB subunit, we speculated that BC biosynthesis may be regulated by QS by controlling c-di-GMP levels. Bacteria generally depend on QS to regulate cellular processes that are essential for survival and adaptation to changing environments. Although it is still unclear why microorganisms produce BC in nature, BC has been shown to confer access to sufficient oxygen at the liquid–gas interface and high resistance to UV irradiation^64^. Therefore, there is a possibility that QS participates in the BC synthesis. Various methods have been applied to manipulate the biosynthesis of BC, but little attention has been paid to the regulation of QS during BC production. Nonetheless, it has been successfully applied to the production of biofilms^65^. The BC-regulatory mechanism involved in the relations among QS, c-di-GMP, and BC is worthy of further study. The regulatory mechanism of action on BC production presented in this study is summarised in Fig. 5.

## Materials and Methods

### Cell culture and chromosomal DNA extraction

*G. xylinus* CGMCC 2955 was isolated from 16 solid fermentation substrates of vinegar by Tianjin University of Science and Technology^18^. After cultivation on a solid medium (25 g/l glucose [0.83 (mol carbon)/l], 10 g/l peptone, 7.5 g/l yeast extract, and 10 g/l Na_2_HPO_4_) at 30 °C for 3 days, cells in the medium were washed with normal saline and centrifuged at 4000 rpm for 5 min (Eppendorf 5804 R). The cell pellets were subjected to DNA extraction. A TAKARA DNA extraction kit was employed for DNA extraction. DNA quality was evaluated on a BASIC biospectrometer (Eppendorf).

### DNA sequencing and assembly

The genome of *G. xylinus* CGMCC 2955 was sequenced at Genewiz Biotechnology Co., Ltd. (Suzhou, China) by means of a PacBio RS II DNA sequencing 9 K library. Single-molecule real-time sequencing (SMRT) allows for highly precise sequencing, with accuracy of more than 99.999% (QV50) and is not affected by GC and AT content. Sequences were assembled according to principles similar to those of the first-generation sequencing technology. Consequently, a single scaffold was assembled in the SMRT Link software (version 4.1). The genomic fine drawing was completed by analysing bioinformatic means after quality control procedures.

### Effects of acetate and ethanol on BC production

*G. xylinus* CGMCC 2955 was cultured as previously reported^3^. When acetate or ethanol was supplied as the sole carbon source, the culture media had the following composition: 0.43 (mol C)/l carbon source, 10 g/l peptone, 7.5 g/l yeast extract, and 10 g/l Na_2_HPO_4_. The initial pH was adjusted to 6.0. The same culture medium without addition of a carbon source served as the control. For supplementary ethanol or acetate experiments, 0.43 (mol C)/l ethanol or acetate was added to the glucose culture medium. BC was harvested after static culture at 30 °C for 10 days. Before weighing, BC was processed as reported elsewhere^3^. The dry weight was recorded for each pellicle at room temperature.

### Nucleotide sequence accession numbers

The complete sequences of *G. xylinus* CGMCC 2955 analysed in this study can be found in the NCBI GenBank (http://www.ncbi.nlm.nih.gov) under accession No. CP024644.

### Biological detection of signalling molecule AHLs

The presence of AHLs in *G. xylinus* CGMCC 2955 was determined in an agar well-diffusion assay and β-galactosidase activity assay based on a biosensor^46^. This is a traditional and powerful method for investigating QS systems in gram-negative bacteria. The AHLs were detected by a vertical streaking agar well diffusion assay with the AHL biosensor.

*G. xylinus* CGMCC 2955 was streaked on Luria Bertani (LB) agar containing 40 μg/ml 5-bromo-4-chloro-3-indolyl β-D-galactopyranoside (X-Gal) perpendicular to *A. tumefaciens* A136, with *P. aeruginosa* PAK serving as the positive control and *A. tumefaciens* A136 alone serving as the negative control. AHL production was detected as the production of a blue pigment by *A. tumefaciens* A136. The AHL biosensor, carrying TraR-regulated *traI-lacZ* fusion genes, can produce a blue pigment in the presence of X-Gal in response to exogenous AHLs.

### Data availability statement

All data generated or analysed during this study are included in this published article.

### Ethical statement

This article does not contain any experiments on human participants or animals that were performed by any of the authors.
